# Systematic vitamin D supplementation is associated with improved outcomes and reduced thyroid adverse events in patients with cancer treated with immune checkpoint inhibitors: results from the prospective PROVIDENCE study

**DOI:** 10.1007/s00262-023-03522-3

**Published:** 2023-08-28

**Authors:** Melissa Bersanelli, Alessio Cortellini, Alessandro Leonetti, Alessandro Parisi, Marcello Tiseo, Paola Bordi, Maria Michiara, Simona Bui, Agnese Cosenza, Leonarda Ferri, Giulia Claire Giudice, Irene Testi, Elena Rapacchi, Roberta Camisa, Bruno Vincenzi, Giuseppe Caruso, Antonio Natale Rauti, Federica Arturi, Marco Tucci, Valentina Santo, Valentina Ricozzi, Vanessa Burtet, Paolo Sgargi, Renata Todeschini, Fable Zustovich, Luigia Stefania Stucci, Daniele Santini, Sebastiano Buti

**Affiliations:** 1 Medical Oncology Unit, University Hospital of Parma, Parma, Italy; 2 Gruppo Oncologico Italiano Di Ricerca Clinica (GOIRC), Parma, Italy; 3 Operative Research Unit of Medical Oncology, Fondazione Policlinico Universitario Campus Bio-Medico, Alvaro del Portillo N° 200, 00128 Rome, Italy; 4 Department of Surgery and Cancer, Hammersmith Hospital Campus, Imperial College London, London, UK; 5 Department of Oncology, Università Politecnica Delle Marche-AOU Delle Marche, 60121 Ancona, Italy; 6 Department of Medicine and Surgery, University of Parma, Parma, Italy; 7 Department of Interdisciplinary Medicine (DIM), University of Bari “Aldo Moro”, Bari, Italy; 8 UOC Farmacia Ospedaliera, Aulss N.1 Dolomiti, Belluno Hospital, Belluno, Italy; 9 Cancer Registry of Parma Province, University Hospital of Parma, Parma, Italy; 10 UOC Oncologia, Aulss N.1 Dolomiti, Belluno Hospital, Belluno, Italy; 11 Medical Oncology Unit, Policlinico Hospital of Bari, Bari, Italy; 12 Oncologia Medica A, Policlinico Umberto 1, La Sapienza Università Di Roma, Rome, Italy

**Keywords:** Immune checkpoint inhibitors, Vitamin D, Cholecalciferol, Immunotherapy, Cancer, Immune related adverse events

## Abstract

**Background:**

Hypovitaminosis D can have a negative prognostic impact in patients with cancer. Vitamin D has a demonstrated role in T-cell-mediated immune activation. We hypothesized that systematic vitamin D repletion could impact clinical outcomes in patients with cancer receiving immune-checkpoint inhibitors (ICIs).

**Methods:**

We planned a prospective observational study (PROVIDENCE) to assess serum vitamin D levels in patients with advanced cancer receiving ICIs (cohort 1 at treatment initiation, cohort 2 during treatment) and the impact of systematic repletion on survival and toxicity outcomes. In an exploratory analysis, we compared the clinical outcomes of cohort 1 with a control cohort of patients followed at the participating centers who did not receive systematic vitamin D repletion.

**Results:**

Overall, 164 patients were prospectively recruited in the PROVIDENCE study. In cohort 1, consisting of 101 patients with 94.1% hypovitaminosis (≤ 30 ng/ml) at baseline, adequate repletion with cholecalciferol was obtained in 70.1% at the three months re-assessment. Cohort 2 consisted of 63 patients assessed for vitamin D at a median time of 3.7 months since immunotherapy initiation, with no patients having adequate levels (> 30 ng/ml). Even in cohort 2, systematic supplementation led to adequate levels in 77.8% of patients at the three months re-assessment.

Compared to a retrospective control group of 238 patients without systematic vitamin D repletion, PROVIDENCE cohort 1 showed longer overall survival (OS,* p* = 0.013), time to treatment failure (TTF,* p* = 0.017), and higher disease control rate (DCR, *p* = *0.016*). The Inverse Probability of Treatment Weighing (IPTW) fitted multivariable Cox regression confirmed the significantly decreased risk of death (HR 0.55, 95%CI: 0.34–0.90) and treatment discontinuation (HR 0.61, 95%CI: 0.40–0.91) for patients from PROVIDENCE cohort 1 in comparison to the control cohort. In the context of longer treatment exposure, the cumulative incidence of any grade immune-related adverse events (irAEs) was higher in the PROVIDENCE cohort 1 compared to the control cohort. Nevertheless, patients from cohort 1 experienced a significantly decreased risk of all grade thyroid irAEs than the control cohort (OR 0.16, 95%CI: 0.03–0.85).

**Conclusion:**

The PROVIDENCE study suggests the potential positive impact of early systematic vitamin D supplementation on outcomes of patients with advanced cancer receiving ICIs and support adequate repletion as a possible prophylaxis for thyroid irAEs.

**Supplementary information:**

The online version contains supplementary material available at 10.1007/s00262-023-03522-3.

## Introduction

Hypovitaminosis D is extremely frequent in patients with cancer, with a recognized negative prognostic impact [1–3]. Calcitriol, or 1,25-dihydroxy-vitamin D3, is a multifunctional steroid hormone with many extra skeletal effects, possibly regulating signaling pathways related to cancer development and progression. Vitamin D promotes cell differentiation and inhibits proliferation, angiogenesis, and cell migration [4]. The multifaceted anti-proliferative effects of Vitamin D involve several pathways, including phosphatidylinositol 3 kinase/AKT, MAPK, NF-kB, and Ca2 + signaling [5]. Vitamin D has been reported to inhibit the cyclin-dependent kinase 2 (CDK2), as well as the insulin like growth factor (IGF)-1- and IGF-2 pathways [5], the Wnt/β-catenin axis [6, 7], and to activate transcription factors like the forkhead box O3/4 (FoxO3/4), which regulates genes involved in cell cycle arrest [8]. In addition, vitamin D also reduces the expression of the telomerase reverse transcriptase (TERT) and increases the expression of transforming growth factor β (TGFβ), along with its receptors, leading to inhibition of cell growth [9, 10].

On the other hand, vitamin D has crucial immunomodulatory effects: the interaction of the active form 1,25(OH)2D or calcitriol with the vitamin D receptor (VDR) is crucial for the proper activation of the immune system, particularly for T cell differentiation and their effector function [11, 12]. Activated T cells can produce 1,25(OH)2D, binding vitamin D-responsive elements (VDRE) and activating vitamin D-responsive gene transcription. These signals upregulate the enzyme phospholipase C-γ1 (PLC-γ1), a key molecule for the classical T cell receptor (TCR) signaling pathway [13]. In turn, antigen-specific triggering of TCR expressed on the surface of naïve T cells has the intracellular effect of promoting the upregulation of the VDR, establishing a virtuous circle of T lymphocyte activation [12]. In addition, 1,25(OH)2D has been shown to affect macrophages (TAMs) in cancer models, reversing M2 polarization of macrophages and their pro-tumorigenic effects on proliferation and migration [14].

It is known that the mechanism of action of ICIs has its target in T-lymphocytes, leveraging on the cell-mediated functions to trigger the anticancer immune response. Interestingly, some previous evidence suggested an interaction between response to ICI and vitamin D metabolism. For example, a decrease in the vitamin D binding protein (DBP), which sequestrates calcidiol and blocks its conversion to calcitriol in T cells [15], was associated with a prolonged overall survival in patients with advanced renal cancer treated with the anti-PD-L1 atezolizumab [16].

Considering the evidence in support of the multifaceted immunological role of vitamin D, we sought to investigate the potential role of hypovitaminosis D and its systematic repletion in patients with cancer treated with ICI-based regimens [17].

## Materials and methods

### Study objectives and design

PROVIDENCE is a prospective observational study aimed at describing baseline serum vitamin D levels and the impact of systematic vitamin D supplementation on clinical outcomes in patients with advanced solid tumors treated with ICIs in clinical practice.

The enrollment was performed at the oncology department of four Italian institutions from November 2017 to January 2020. Patients with advanced solid tumors candidates to receive immunotherapy who had not been treated with vitamin D supplementation over the 12 months before enrollment were recruited at ICI initiation (Cohort 1) or during treatment (Cohort 2) for the vitamin D serum level assessment. In Cohort 1, vitamin D was assessed within 30 days before treatment initiation; in Cohort 2, it was evaluated at the time of accrual. Cohort 2 was an "ethical" cohort, established in view of the study hypothesis, which assumed an immune-modulating effect of Vitamin D, in order to provide the same potential benefit to all patients treated with ICI at the participating centers during the study period.

All centers (the University Hospitals of Parma, L’Aquila, and Bari, and the Hospital of Belluno) participated in cohort 2, while only two centers enrolled patients in cohort 1 (the University Hospitals of Parma and L’Aquila). In each cohort, patients were stratified according to the serum vitamin D levels into adequate level group (> 30 ng/ml), insufficiency group (> 20–30 ng/ml), deficiency group (> 10–20 ng/ml), and severe deficiency group (≤ 10 ng/ml).

At enrollment, vitamin D supplementation was systematically offered to patients starting within 28 days from the assessment, following the guidelines of the Italian Society of Osteoporosis, Mineral Metabolism, and Bone Disease (SIOMMMS [18]) as follows:

- Adequate level group: no supplementation.
- Insufficiency group: cholecalciferol (vitamin D3) at the loading dose of 300,000 International Units (IU) over 4 weeks, maintenance dose of 820 IU daily.
- Deficiency group: cholecalciferol at the loading dose of 600,000 International Units (IU) over 4 weeks, maintenance dose of 1000 IU daily.
- Severe deficiency group: cholecalciferol at the loading dose of 1,000,000 International Units (IU) over 4 weeks, maintenance dose of 2000 IU daily.

Serum vitamin D levels were subsequently assessed every 12 weeks (± 1 week) up to 9 months from treatment initiation among alive patients at each landmark point. Supplementation was continued at least until ICI treatment failure or terminated according to clinical practice in the case of hypervitaminosis.

Being a real-world study, clinical outcomes of interest included time to treatment failure (TTF), defined as the time from treatment initiation to treatment discontinuation for whatever cause, including toxicity and disease progression, and overall survival (OS), defined as the time from treatment initiation to patients’ death or loss to follow-up. We also assessed objective response rate (ORR), defined as the proportion of patients achieving partial/complete response as best response to treatment, and disease control rate (DCR), defined as the proportion of patients achieving partial/complete response or stable disease as best response. Periodic tumor re-assessment was performed at the discretion of treating clinicians with frequency ranging from 12 to 16 weeks; investigators were asked to provide disease assessments following Response Evaluation Criteria in Solid Tumors (RECIST) criteria v1.1. TTF and OS were measured from treatment initiation to treatment discontinuation or death, respectively. Patients without documented discontinuation/death were censored on the date of the last clinical follow-up. The data cut-off date was October 2020.

We also evaluated the cumulative incidence of immune-related adverse events (irAEs) of all grades and grade 3/grade 4 (G3/G4) irAEs in Cohort 1. Immune-related AEs were categorized based on the organ/system involved into thyroid irAEs, other endocrine irAEs, colitis, cutaneous irAEs, pneumological irAEs, hepatic irAEs, rheumatologic irAEs and neuro-muscular irAEs. Adverse events were assessed by treating clinicians in clinical practice according to the National Cancer Institute Common Toxicity Criteria for Adverse Events (CTCAE) version 4.0 [19].

Considering the very high prevalence of hypovitaminosis D that we found in PROVIDENCE Cohort 1, we used a retrospective control cohort made of patients with advanced solid tumors treated in clinical practice with ICI-regimens (without chemotherapy and/or targeted therapy) at the two participating centers that enrolled patients into cohort 1 (University Hospitals of Parma and L’Aquila), to explore the potential positive impact on clinical outcomes of adequate systematic vitamin D supplementation.

The control cohort included consecutive patients who started immunotherapy before the PROVIDENCE enrollment period with unknown baseline vitamin D serum levels, from December 2014 to January 2020, with September 2020 as data cut-off period.

Therefore, the control cohort consisted of patients with baseline unknown vitamin D levels, assuming a high prevalence of hypovitaminosis D. However, patients who started ICI therapy during the PROVIDENCE enrollment period, but where ineligible to enter cohort 1 because of prior vitamin D supplementation (at any dose and for any reason) during the previous 12 months, were included in the control cohort, whilst patients who have been subsequently enrolled in PROVIDENCE cohort 2 have been excluded. In summary, the control cohort was strictly characterized by having never received systematic vitamin D supplementation as described above for the PROVIDENCE cohorts.

Considering that patients subsequently enrolled in PROVIDENCE cohort 2 were positively selected for treatment duration, possibly leading to an underestimation of survival outcomes in the control cohort with their exclusion, we performed an additional explorative comparative analysis, including them in the control group.

**Fig. 1:**
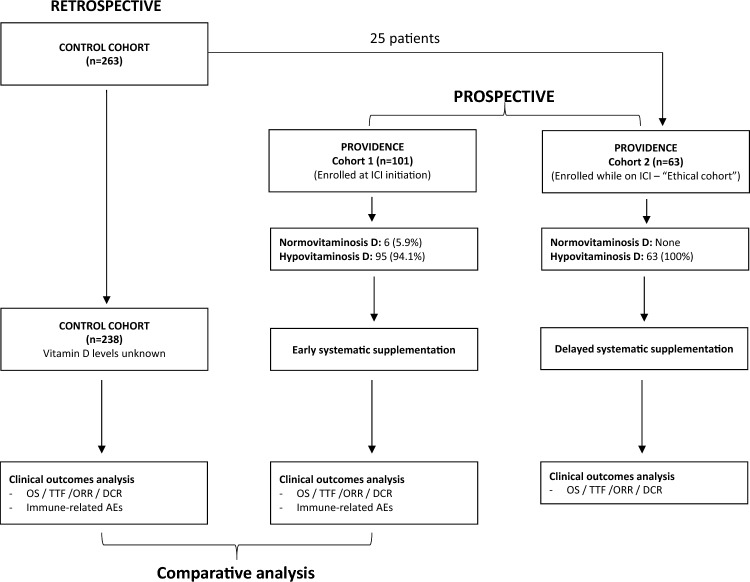
Study flow diagram

A study flow diagram is provided as Fig. 1. Eligibility criteria for PROVIDENCE cohort 1 and cohort 2 and for the control cohort were as follows: confirmed histological diagnosis of solid malignancy, advanced stage disease (stage IV), the receipt of ICI regimens outside of interventional clinical trials, age ≥ 18 years and written informed consent.

A detailed description of statistical analysis is provided as supplementary methods.

## Results

### Cohort 1

Overall, 101 patients were included in the PROVIDENCE Cohort 1 (Table 1). Administered ICIs were nivolumab (45, 44.6%), pembrolizumab (37, 36.6%), atezolizumab (10, 9.9%), avelumab (2, 2.0%), cemiplimab (4, 4.0%) and nivolumab/ipilimumab combination (3, 3.0%). Most patients were male (77, 76.2%), aged ≥ 70 years (55, 54.5%), with ≤ 2 metastatic sites (66, 65.3%), and treated in second/further line settings (54, 53.5%), with NSCLC being the most frequent primary tumor (49.5%).

**Table 1:** Baseline patients’ characteristics for the PROVIDENCE cohort 1, cohort 2 and for the control cohort. ECOG-PS: eastern cooperative oncology group-performance status; NSCLC: non-small cell lung cancer

	PROVIDENCE Cohort 1	PROVIDENCE Cohort 2	Control cohort
	N°(%)–101	N°(%)–63	N°(%)–238
*Age (years)*			
Median	71	71	69
Range	39–90	36–87	28–98
Elderly (≥ 70 years)	55(54.5)	32(50.8)	55(54.5)
*Sex*			
Female	24(23.8)	32(50.8)	81(44.0)
Male	77(76.2)	31(49.2)	157(66.0)
*ECOG–PS*			
0	46(45.5)	9(14.3)	78(32.8)
1	44(43.6)	16(25.4)	105(44.1)
≥ 2	11(10.9)	3(4.8)	55(23.1)
Unknown	–	35(55.6)	–
*Primary Tumor*			
NSCLC	50(49.5)	26(41.3)	48(20.2)
Melanoma	27(26.7)	34(54.0)	37(15.5)
Renal cell carcinoma	13(12.9)	1(1.6)	125(52.5)
Urothelial	4(4.0)	2(3.2)	18(7.6)
Others	7(6.9)	–	10(4.2)
*No. of metastatic sites*			
≤ 2	66(65.3)	–	112(47.1)
> 2	35(34.7)	–	126(52.9)
*Treatment line of Immunotherapy*			
First	47(46.5)	32(50.8)	49(22.1)
Non–First	54(53.5)	31(49.2)	189(79.4)
*Baseline Vitamin D (ng/ml)*			
Median (range)	13(4–73)	11(4–29)	–
Adequate (> 30)	6(5.9)	–	–
Insufficiency (20–30)	23(22.8)	12(19.0)	–
Deficiency (10–20)	39(38.6)	24(38.1)	–
Severe deficiency (< 10)	33(32.7)	27(42.9)	–
*Immunotherapy*			
Nivolumab	45(44.6)	–	–
Pembrolizumab	37(36.6)	27(42.9)	–
Atezolizumab	10(9.9)	5(7.9)	–
Avelumab	2(2.0)	–	–
Cemiplimab	4(4.0)	–	–
Nivolumab–Ipilimumab	3(3.0)	–	–

Notably, only 6 patients (5.9%) had an adequate baseline vitamin D level, while 22.8%, 38.6%, and 32.7% presented with insufficiency, deficiency, and severe deficiency, respectively. We found no association between baseline vitamin D levels (adequate *vs*. non-adequate) and age (*p* = 0.8221), patients’ sex (*p* = 0.6752), ECOG-PS (*p* = 0.9288), number of metastatic sites (*p* = 0.9444), treatment line (*p* = 0.3104), and primary tumor (*p* = 0.9288).

**Fig. 2:**
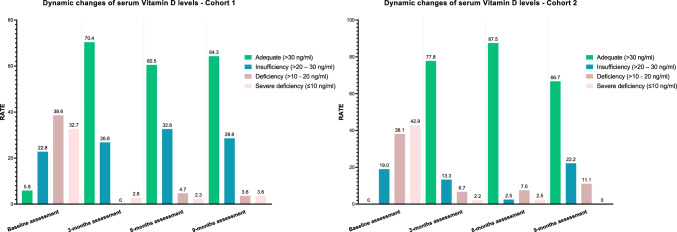
Summary of baseline and dynamic changes over time of serum vitamin D levels in Cohort 1 and Cohort 2. Details are provided as Supplementary Table 1

Overall, supplementation protocols led to the improvement of vitamin D levels, with 70.4%, 60.5%, and 64.3% of patients showing adequate levels (> 30 ng/ml) at the 3-months, 6-months, and 9-months landmark points, respectively (Fig. 2 and Supplementary Table 1).

**Fig. 3:**
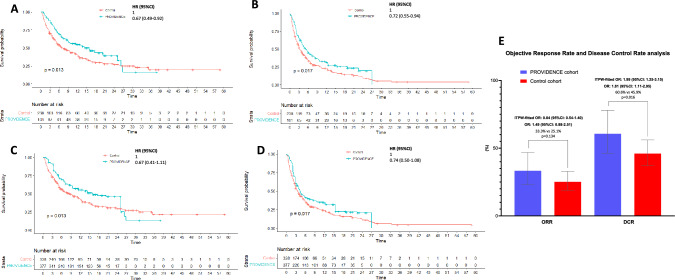
Kaplan–Meier survival estimates with log-rank *p* value and univariable hazard ratio corrected following center-specific conditional interpretation. **A** Overall Survival; PROVIDENCE cohort 1: 15.9 months (95%CI: 8.9—26.8, 52 events), control cohort: 7.1 months (95%CI: 5.3 – 11.0, 155 events). **B** Time to treatment failure; PROVIDENCE cohort 1: 5.2 months (95%CI: 3.9—7.9, 74 events), control cohort: 3.1 months (95%CI: 2.7 – 4.2, 199 events). **C** IPTW-fitted Overall Survival; PROVIDENCE cohort 1: 15.9 months (95%CI: 8.8—NR), control cohort: 8.5 months (95%CI: 5.6 – 12.1). **D** IPTW-fitted time to treatment failure; PROVIDENCE cohort 1: 4.3 months (95%CI: 3.1—5.6), control cohort: 3.5 months (95%CI: 2.9 – 4.8). **E)** Objective Response Rate and Disease Control Rate analysis. ORR and DCR are reported as crude rates with 95%CI. Univariable and IPTW-fitter OR with 95%CI were computed using logistic regression. IPTW: inverse probability of treatment weighing, NR: not reached, HR: hazard ratio; CI: confidence intervals; OR: odds ratio; ORR: objective response rate; DCR: disease control rate

At the median follow-up of 15.4 months (95%CI: 13.8–17.0), the median OS of the PROVIDENCE cohort 1 was 15.9 months (95%CI: 8.9–26.8, 52 events), while the median TTF was 5.2 months (95%CI: 3.9–7.9; 74 events) (Fig. 3A and Fig. 3B). Among evaluable patients for disease response, the ORR and the DCR were 33.3% (95%CI: 22.9–46.8) and 60.6% (95%CI: 46.2–78.1), respectively (Fig. 3E). Overall, 60 patients (59.4%) experienced any-grade irAEs, while 13 patients (12.9%) experienced G3/G4 irAEs. The most frequently reported irAEs of any grade were colitis (22.8%) and cutaneous irAEs (18.8%), with only 4% of patients reporting any grade thyroid irAEs.

### Cohort 2

Four centers enrolled patients into the PROVIDENCE cohort 2 (University Hospitals of Parma, L’Aquila, and Bari, and Hospital of Belluno), consisting of 63 patients (Table 1). In this Cohort, the median time from ICI treatment initiation to vitamin D level assessment was 3.7 months (range: 0.7–57.4) with a median value of 11 ng/ml. There were no patients with adequate vitamin D levels at baseline, and 19.0%, 38.1%, and 42.9% of patients had insufficiency, deficiency, and severe deficiency, respectively, with no association with the available clinical features.

Even in cohort 2, vitamin D supplementation led to meaningful improvements in vitamin D levels, with 77.8%, 87.5%, and 66.7% of patients showing adequate levels (> 30 ng/ml) at the 3-months, 6-months, and 9-months landmark points, respectively (Fig. 2 and Supplementary Table 1). At the median follow-up of 32.5 months (95%CI: 22.8–38.1), the median OS of cohort 2 was not reached (23 events), and the median TTF was 23.2 months (95%CI: 13.1–28.8, 40 events). Among evaluable patients for disease response the ORR was 33.3% (95%CI: 20.4–51.5) and the DCR was 81.7% (95%CI: 60.4–99.9).

### Control cohort

The control cohort was gathered from the same two institutions that enrolled patients into PROVIDENCE cohort 1. After the exclusion of 25 patients who were subsequently enrolled into PROVIDENCE cohort 2, the control cohort consisted of 238 patients, of whom 111 (46.6%) aged ≥ 70 years, 157 (66.0%) males, 126 (52.9%) with > 2 metastatic sites, 189 (79.4%) being treated in the advanced line setting, and 105 (44.1%) and 55 (23.1%) with a baseline ECOG-PS of 1 and ≥ 2, respectively (Table 1). Most patients had advanced renal cell carcinoma (n = 125, 52.5%), and the median follow-up period for the cohort was 19.3 months (95%CI: 14.9–25.1).

### Comparison of outcomes between cohort 1 and control cohort

The median OS and TTF for the control group were 7.1 months (95%CI: 5.3 – 11.0, 155 events) and 3.1 months (95%CI: 2.7–4.2, 199 events), which were significantly shorter when, respectively, compared to those of the PROVIDENCE cohort 1 (log-rank *p* value 0.013, HR for the risk of death 0.67 [95%CI: 0.49–0.92] Fig. 3A; log-rank *p* value 0.017, HR for the risk of treatment discontinuation 0.72 [95%CI: 0.55–0.94] Fig. 3B). Patients from the control cohort achieved an ORR of 25% (95%CI: 18.8–32.9), which was similar to that of the PROVIDENCE cohort 1 (OR 1.49, 95%CI: 0.88–2.52). On the other hand, the DCR of 45.9% (95%CI: 37.1–56.1) achieved by the control cohort was significantly lower than that reported for the PROVIDENCE cohort 1 (OR 1.81, 95%CI: 1.11–2.95) (Fig. 3E).

Supplementary Table 2 reports the pre- and post-weighing distribution of baseline characteristics; the balancing ability was suboptimal (SMD ≥ 0.1) for ECOG-PS, number of metastatic sites, and primary tumor. The Inverse Probability of Treatment Weighing (IPTW) fitted univariable analysis confirmed the longer OS (log-rank *p* value 0.013, Fig. 2C) and TTF (log-rank *p* value 0.017, Fig. 2D) for the PROVIDENCE cohort 1. The IPTW-fitted comparisons did not confirm a significantly decreased risk of death (HR 0.67, 95%CI: 0.41–1.11) and treatment discontinuation (HR 0.74, 95%CI: 0.50–1.08), nor increased probability of achieving disease response (OR 0.84, 95%CI: 0.54–1.40), in comparison to the control cohort, whilst increased DCR for the PROVIDENCE cohort 1 found further confirmation (OR 1.99, 95%CI: 1.25–3.15) (Fig. 3E). IPTW-fitted multivariable Cox regression, including ECOG-PS, number of metastatic sites, and primary tumor, showed that patients from the PROVIDENCE cohort 1 experienced a significantly decreased risk of death (HR 0.55, 95%CI: 0.34–0.90), and treatment discontinuation (HR 0.61, 95%CI: 0.40–0.91) (Supplementary Table 3) when compared to the non-repleted control cohort.

To mitigate the negative selection caused by the exclusion of patients subsequently enrolled into PROVIDENCE cohort 2, we performed an additional IPTW-fitted multivariable analysis for treatment discontinuation and death risk using the whole control cohort. Supplementary Table 4 reports the pre- and post-weighing distribution of baseline characteristics of cohort 1 and the full control cohort of 263 patients. Supplementary Table 5 reports the IPTW-fitted multivariable Cox regression, including ECOG-PS and primary tumor, which confirmed that patients from the PROVIDENCE cohort 1 experienced a significantly decreased risk of death (HR 0.62, 95%CI: 0.39–0.98) and treatment discontinuation (HR 0.68, 95%CI: 0.47–0.98).

Table 2 summarizes cumulative rates of irAEs of all grades and G3/G4 irAEs for both the PROVIDENCE cohort 1 and the control cohort, along with the relevant adjusted OR, including the interaction between exposure to treatment (i.e., TTF) and systematic vitamin D supplementation (i.e., the cohort). In the context of a prolonged treatment exposure reported for cohort 1, numerically higher rates of any grade and G3/G4 irAEs were reported for the PROVIDENCE cohort compared to the control cohort, except for thyroid irAEs. The multivariable analysis confirmed a significantly higher probability of experiencing overall G3/G4 irAEs (OR 3.21, 95%CI: 1.21–8.56) for Cohort 1. On the contrary, patients receiving vitamin D supplementation (cohort 1) were confirmed to experience a significantly lower risk of all grade thyroid irAEs than the control cohort (OR 0.16, 95%CI: 0.03–0.85).

**Table 2:** Summary of all-grade and G3/G4 irAEs rates between the PROVIDENCE cohort 1 and the control cohort. In between cohorts comparisons were presented with univariable Chi-squared test. Separate multivariable logistic regressions including the interaction between exposure to treatment (i.e., TTF) and vitamin D systematic supplementation (i.e., the cohort) were performed to compute the OR with 95%CIs for the risk of adverse events. irAEs: immune-related adverse events; aOR: adjusted odd ratio, 95%CI: 95% confidence interval; TTF: time to treatment failure; TTF*cohort: interaction between TTF and the cohort

	irAEs of any grade				G3/G4 irAEs			
	N°(%)			aOR(95%CI)–TTF*cohort INTERACTION *p* value	N°(%)			aOR(95%CI)–TTF*cohort INTERACTION *p* value
	Cohort 1	Control	*p* value		Cohort 1	Control	*p* value	
Patients	101	238			101	238		
Overall	60(59.4)	91(38.2)	0.001	1.16(0.60–2.25)–0.005	13(12.9)	15(6.3)	0.045	3.21(1.21–8.56)–0.2670
Thyroid irAEs	4(4.0)	33(13.9)	0.008	0.16(0.03–0.85)–0.426	2(2.0)	–	0.029	–
Other endocrine irAEs	2(1.9)	1(0.4)	0.161	9.48(0.59–152.43)–0.481	2(1.9)	1(0.4)	0.158	9.62(0.60–154.13)–0.477
Colitis	23(22.8)	27(11.3)	0.007	1.29(0.55–3.03)–0.032	4(4.0)	4(1.7)	0.206	3.69(0.66–20.54)–0.482
Cutaneous irAEs	19(18.8)	30(12.6)	0.138	0.53(0.19–1.43)–0.001	1(1.0)	1(0.4)	0.531	3.26(0.11–97.32)–0.781
Pneumological irAEs	6(5.9)	6(2.5)	0.119	1.48(0.31–7.13)–0.285	2(1.9)	5(2.1)	0.943	1.77(0.21–15.16)–0.503
Hepatic irAEs	5(5.0)	5(2.1)	0.157	0.78(0.11–5.48)–0.050	1(1.0)	2(0.8)	0.893	0.85(0.02–25.40)–0.763
Rheumatologic irAEs	4(4.0)	9(3.8)	0.937	0.52(0.08–3.31)–0.234	–	–	–	–
Neuro-muscular irAEs	2(1.9)	4(1.7)	0.848	0.72(0.06–8.74)–0.531	–	–	–	–

## Discussion

To the best of our knowledge, this is the first study prospectively describing baseline vitamin D levels in a cohort of patients with advanced cancer treated with ICIs, providing practice informative evidence about the prevalence of hypovitaminosis D and the possible positive impact of systematic supplementation in this setting.

The first result to consider is the very high prevalence of hypovitaminosis in this population, as the vast majority of the enrolled patients (> 90%) had non-adequate (< 30 ng/ml) vitamin D serum levels, a finding that is in line with epidemiological data about hypovitaminosis D, known to be endemic in Italy in healthy subjects, especially in the geographic areas of recruitment [20]. In addition, literature data suggest the existence of complex, two-way relationship between Vitamin D metabolism and cancer, from the one hand Vitamin D inadequate levels can be seen as a risk factor for developing cancer, from the other hand cancer may impact calcitriol levels through peripheral regulation mechanisms that are deranged in many cancer cells [21].

Evidence in melanoma supports the negative prognostic impact of baseline hypovitaminosis D and inadequate correction over time [3], while in our systematically repleted population, most patients reached adequate levels within the first re-assessment. On the contrary, the control cohort did not receive any supplementation, and although consisting of patients with unknown vitamin D levels, we can assume for this group a high prevalence of baseline hypovitaminosis, at least similar to that reported for cohort 1. Our findings in terms of OS, TTF, DCR and immune-related toxicity seem consistent with the initial hypothesis that subjects with adequate vitamin D serum levels may experience an enhanced immune activation, resulting in improved disease control and prolonged survival but also in a higher incidence of irAEs, which however, needs to be interpreted in the context of a longer exposure to ICIs for the PROVIDENCE cohort 1, with the relevant immortal time bias. In connection with our efficacy results, Galus et al., recently described improved response rates among patients with melanoma who maintained adequate levels of vitamin D during PD-1 inhibition [22], further corroborating our findings.

Despite the inclusion of the interaction *p* value between treatment exposure and vitamin D as adjusting factor, the higher risk of overall G3/G4 irAEs reported for the PROVIDENCE cohort 1 in comparison to the control group, may have been flawed by residual patients’ selection towards increased cumulative risk. This factor may have also influenced the numerically higher incidence of any grade and G3/G4 colitis among vitamin D recipients, a finding that conflicts with the increasing evidence supporting the role of vitamin D in modulating the intestinal microbiota, resulting in improved barrier permeability and decreased inflammation in inflammatory bowel disease [23].

In this context, the significantly decreased risk of thyroid irAEs for the PROVIDENCE cohort in comparison to the control group is undoubtedly suggestive and suggest systematic vitamin D supplementation as prophylactic treatment for immune-related thyroid disorders during ICI-based treatments.

Thyroid irAEs are based on T-cell-mediated autoimmune reactions, as well as primitive autoimmune thyroid disorders [24, 25]. Interestingly, polymorphisms in VDR and other genes involved in vitamin D dependent signaling were demonstrated to be associated with an increased risk of autoimmune thyroid diseases [26]. In addition, three comprehensive meta-analyses showed that vitamin D deficiency is associated with autoimmune thyroid disorders, including Hashimoto’s thyroiditis, and hypothyroidism [27–29], while experimental studies showed that vitamin D directly affects Dio2, the enzyme which drives the T4/T3 conversion in target tissues [30]. Several reports indicate that vitamin D deficiency may contribute to autoimmunity via its effects on the intestinal barrier function, microbiome composition, and/or direct effects on immune responses [31], and prospective evidence suggest that high-dose vitamin D3 can significantly reduce CD4 + T-cell activation compared to low-dose vitamin D3 [32].

Our study acknowledges several limitations, mainly related to the observational design, the limited sample size of subgroups and the post hoc approach used for obtaining the control group, which was adopted because of the high prevalence of hypovitaminosis found in PROVIDENCE cohort 1. This impaired our ability of performing any comparative subgroup analysis within the same population. Despite the strict statistical methodology using double-adjusted IPTW-fitted models with center-specific correction of 95%CIs, which allowed us to obtain comparable cohorts, the differential distribution of baseline characteristics between the two cohorts, such as the proportion of different primary tumors, needs to be taken in to account, along with the shorter follow-up period for PROVIDENCE cohort 1. Lastly, we need to mention the lack of baseline information possibly related to vitamin D levels and metabolism, such as body mass index and other body composition measures, other concomitant medications and dietary habits [33].

Despite the mentioned limitations, our study provides for the first-time practice informing evidence about the high prevalence of hypovitaminosis D in patients with solid tumors treated with ICI and on the efficacy of systematic supplementation to timely restore adequate vitamin D levels in most patients. Our explorative comparative analysis offers provocative insights about the putative multifaceted immune-modulating effects of vitamin D systematic supplementation, which could potentially improve clinical outcomes and prevent thyroid irAEs in patients receiving ICI-based treatments. However, properly powered comparative studies are still needed to confirm our findings along with comprehensive researches to fully elucidate the underlying mechanism involved.

### Supplementary information

Below is the link to the electronic supplementary material.

Supplementary file1 (DOCX 46 KB)

## Data Availability

De-identified participant data and data dictionary may be made available at reasonable request of investigators whose proposed use of the data has been approved by the authors and local ethical committee.
