# Supplementary material for: Systematic vitamin D supplementation is associated with improved outcomes and reduced thyroid adverse events in patients with cancer treated with immune checkpoint inhibitors: results from the prospective PROVIDENCE study
Source: Cancer Immunol Immunother. 2023 Aug 28;72(11):3707–16. doi: 10.1007/s00262-023-03522-3 (PMC10576732; doi:10.1007/s00262-023-03522-3)
Supplement: Supplementary file 1 — Supplementary file1 (DOCX 46 KB) [file 262_2023_3522_MOESM1_ESM.docx]

**Systematic vitamin D supplementation is associated with improved outcomes and reduced thyroid adverse events in patients with cancer treated with immune checkpoint inhibitors: results from the prospective PROVIDENCE study.**

Bersanelli M et al.

**Contents**

**Supplementary Methods Page 2**

**Supplementary Table 1 Page 4**

**Supplementary Table 2** **Page 5**

**Supplementary Table 3** **Page 5**

**Supplementary Table 4** **Page 6**

**Supplementary Table 5** **Page 6**

**Supplementary Methods**

**Statistical analysis**

Baseline patients’ characteristics were reported with descriptive statistics as appropriate. The χ2 test was used to compare categorical variables.

Considering that cumulative incidence of adverse events during treatment is time-dependent, the probability of experiencing each irAE category between the PROVIDENCE Cohort 1 and the control cohort was compared with multivariable logistic regressions including the interaction term between the cohort and treatment duration (TTF) and estimated through adjusted odd ratios (OR) with 95% confidence intervals (CIs)

Median TTF and overall survival (OS) were evaluated using the Kaplan-Meier method and compared with the log-rank test. Objective response rate (ORR) and disease control rate (DCR) were reported as crude rates with 95%CI. The duration of follow-up was calculated according to the reverse Kaplan-Meier method. Considering the limited sample size of subgroups, an Inverse Probability of Treatment Weighing (IPTW) procedure was used to weigh key baseline characteristics between the PROVIDENCE cohort 1 and the control cohort to fit comparative univariable analysis, with balancing ability estimated through the standardized mean differences (SMD) of the weighted characteristics. Key variables included: primary tumor types (non-small cell lung cancer - NSCLC, melanoma, renal cell carcinoma, urothelial cancers, and others), age (≥ *vs.* < 70 years), biological sex (male *vs.* female), Eastern Cooperative Oncology Group-Performance Status (ECOG-PS) (0 vs. 1 *vs*. ≥ 2), burden of disease (number of metastatic sites ≤ 2 *vs*. > 2), treatment line (first *vs.* second and further lines of therapy).

Cox proportional hazards regression was used to estimating the risk of treatment discontinuation/death and presented through hazard ratios (HR) with 95%CIs. To further mitigate any residual imbalance of key characteristics, we also performed a double adjustment, including variables with post-weighing SMD ≥0.10 in IPTW-fitted multivariable regression models for the risk of treatment discontinuation (TTF) and death (OS) [14]. Probability of achieving objective response (ORR) and disease control (DCR) were also compared with logistic regression and presented through OR with 95%CI.

Acknowledging that the data source consisted of different institutions, with patients followed by treating physicians in clinical practice, therefore without pre-established monitoring procedures, a clustered-robust correction for participating center was applied to 95%CI from logistic regression and a center-specific conditional interpretation by using frailty models was applied to correct all the 95%CIs from multivariable Cox regressions, whilst a clustered-robust correction for participating center was applied to 95%CI from multivariable logistic regressions. All P-values were 2-sided, and confidence intervals were set at the 95% level, with significance pre-defined to be at <0.05. Analyses were performed using the R-studio software, R Core Team (2021). R: A language and environment for statistical computing. R Foundation for Statistical Computing, Vienna, Austria, and the MedCalc® Statistical Software version 20 (MedCalc Software Ltd, Ostend, Belgium; https://www.medcalc.org; 2021).

**Supplementary Table 1:** Summary of dynamic changes in Vitamin D levels over time in cohorts 1 and 2.

|  | **Baseline** | **3-months** | **6-months** | | **9-months** | |
| --- | --- | --- | --- | --- | --- | --- |
| **Vitamin D** | **N° (%) – 101** | **N° (%) – 71** | **N° (%) – 43** | | **N° (%) – 28** | |
| **Cohort 1** | | | | | | |
| **Median ng/ml (range)**  Adequate (>30)  Insufficiency (20 - 30)  Deficiency (10 -20)  Severe deficiency (<10) | **13 (4 – 73)**  6 (5.9)  23 (22.8)  39 (38.6)  33 (32.7) | **38 (5 – 95)**  50 (70.4)  19 (26.8)  -  2 (2.8) | **31 (7 – 106)**  26 (60.5)  14 (32.6)  2 (4.7)  1 (2.3) | | **34 (6 – 59.3**)  18 (64.3)  8 (28.6)  1 (3.6)  1 (3.6) | |
| **Cohort 2** | | | | | | |
|  | **n = 63** | **n = 45** | | **n = 40** | | **n = 36** |
| **Median (ng/ml) (range)**  Adequate (>30)  Insufficiency (20 - 30)  Deficiency (10 -20)  Severe deficiency (<10) | **11 (4 – 29)**  -  12 (19.0)  24 (38.1)  27 (42.9) | **41 (8-125)**  35 (77.8)  6 (13.3)  3 (6.7)  1 (2.2) | **36 (9-77)**  35 (87.5)  1 (2.5)  3 (7.5)  1 (2.5) | | **33 (10-56)**  24 (66.7)  8 (22.2)  4 (11.1)  - | |

**Supplementary Table 2:** Comparison of baseline patients’ characteristics between the PROVIDENCE cohort 1 and the control cohort before and after the ITPW procedure. ECOG-PS: eastern cooperative oncology group-performance status; NSCLC: non-small cell lung cancer; SMD: standardized mean difference; IPTW: inverse probability of treatment weighing.

|  | **PROVIDENCE**  **Cohort 1** | **Control cohort** |  | **PROVIDENCE**  **Cohort 1**  **Weighted** | **Control cohort**  **Weighted** |  |
| --- | --- | --- | --- | --- | --- | --- |
|  | **N° (%) – 101** | **N° (%) – 238** | **p-value - SMD** | **%** | **%** | **p-value - SMD** |
| **Age, (years)**  Non-elderly  Elderly (≥70 years) | 46 (45.5)  55 (54.5) | 127 (53.4)  111 (46.6) | 0.231 – 0.15 | 48.2  51.8 | 50.4  49.6 | 0.817 – 0.04 |
| **Sex**  Female  Male | 24 (23.8)  77 (76.2) | 81 (44.0)  157 (66.0) | 0.081 – 0.22 | 31.0  69.0 | 32.1  67.9 | 0.895 – 0.02 |
| **ECOG-PS**  0  1  ≥ 2 | 46 (45.5)  44 (43.6)  11 (10.9) | 78 (32.8)  105 (44.1)  55 (23.1) | 0.014 – 0.36 | 36.2  34.0  29.7 | 37.0  43.4  19.7 | 0.379 – 0.25 |
| **Primary Tumor**  NSCLC  Melanoma  Renal cell carcinoma  Urothelial  Others | 50 (49.5)  27 (26.7)  13 (12.9)  4 (4.0)  7 (6.9) | 48 (20.2)  37 (15.5)  125 (52.5)  18 (7.6)  10 (4.2) | <0.001 – 1.02 | 27.4  13.6  50.2  3.9  4.9 | 29.6  17.2  41.0  6.4  5.7 | 0.653 – 0.20 |
| **No. of metastatic sites**  ≤ 2  > 2 | 66 (65.3)  35 (34.7) | 112 (47.1)  126 (52.9) | 0.003 – 0.37 | 45.6  54.4 | 52.6  47.4 | 0.424 – 0.14 |
| **Treatment line of Immunotherapy**  First  Non-First | 47 (46.5)  54 (53.5) | 49 (22.1)  189 (79.4) | <0.001 – 0.57 | 24.6  75.4 | 28.4  71.6 | 0.548 – 0.08 |

**Supplementary Table 3:** IPTW-fitted multivariable analysis for the risk of treatment discontinuation and risk of death including variables with SMD ≥0.1. A centre-specific conditional interpretation by using frailty models was applied to correct all the 95%CIs for HR and a clustered robust correction for participating center was applied to correct all the 95%CI for OR. HR: hazard ratio; NSCLC: non-small cell lung cancer; ECOG-PS: eastern cooperative oncology group performance status; IPTW: inverse probability of treatment weighing; SMD: standardized mean difference.

|  | **Multivariate Analysis** | | | |
| --- | --- | --- | --- | --- |
|  | **Risk of Treatment discontinuation** | **Risk of death** | **Probability of achieving tumour response** | **Probability of achieving disease control** |
| **VARIABLE** | **HR (95% CI)** | **HR (95%CI)** | **OR (95% CI)** | **OR (95%CI)** |
| **Cohort**  Control  PROVIDENCE cohort 1 | 1  **0.61 (0.40-0.91)** | 1  **0.55 (0.34-0.90)** | 1  0.89 (0.40-2.00) | 1  1.95 (0.84-4.31) |
| **ECOG-PS**  0  1  ≥2 | 1  1.98 (1.39-2.82)  3.68 (2.11-6.41) | 1  2.34 (1.47-3.73)  3.03 (1.32-6.98) | 1  0.82 (0.37-1.82)  1.15 (0.44-2.98) | 1  0.85 (0.50-1.41)  1.61 (0.43-5.91) |
| **Primary Tumour**  NSCLC  Melanoma  Kidney  Urothelial  Others | 1  0.95 (0.64-1.40)  0.66 (0.43-1.02)  0.69 (0.37-1.27)  0.68 (0.30-1.52) | 1  0.81 (0.51-1.26)  0.61 (0.35-1.04)  0.68 (0.33-1.42)  0.74 (0.29-1.89) | 1  1.06 (0.43-2.62)  0.43 (0.03-5.84)  0.45 (0.03-8.61)  2.35 (0.74-7.40) | 1  1.57 (0.88-2.81)  1.61 (0.55-4.69)  0.85 (0.18-3.98)  2.45 (0.82-7.28) |
| **Number of metastatic sites**  ≤ 2  >2 | 1  1.35 (1.03-1.75) | 1  1.37 (0.90-2.10) | 1  0.79 (0.47-1.31) | 1  0.56 (0.28-1.09) |

**Supplementary Table 4:** Comparison of baseline patients’ characteristics between the PROVIDENCE and the control cohort before and after the ITPW procedure. Patients subsequently entered into PROVIDENCE cohort 2 are included in the control cohort. ECOG-PS: eastern cooperative oncology group-performance status; NSCLC: non-small cell lung cancer; SMD: standardized mean difference; IPTW: inverse probability of treatment weighing.

|  | **PROVIDENCE**  **Cohort 1** | **Control cohort** |  | **PROVIDENCE**  **Cohort 1**  **Weighted** | **Control cohort**  **Weighted** |  |
| --- | --- | --- | --- | --- | --- | --- |
|  | **N° (%) – 101** | **N° (%) – 263** | **p-value - SMD** | **%** | **%** | **p-value - SMD** |
| **Age, (years)**  Non-elderly  Elderly (≥70 years) | 46 (45.5)  55 (54.5) | 139 (52.9)  124 (47.1) | 0.258 – 0.14 | 45.5  54.5 | 50.7  49.3 | 0.989 – 0.01 |
| **Sex**  Female  Male | 24 (23.8)  77 (76.2) | 91 (34.6)  172 (65.4) | 0.062 – 0.24 | 34.1  65.9 | 32.3  67.7 | 0.826 – 0.03 |
| **ECOG-PS**  0  1  ≥ 2 | 46 (45.5)  44 (43.6)  11 (10.9) | 92 (35.0)  116 (44.1)  55 (20.9) | 0.045 – 0.30 | 40.5  33.7  25.8 | 38.4  43.3  18.3 | 0.428 – 0.22 |
| **Primary Tumor**  NSCLC  Melanoma  Renal cell carcinoma  Urothelial  Others | 50 (49.5)  27 (26.7)  13 (12.9)  4 (4.0)  7 (6.9) | 60 (22.8)  40 (15.2)  133 (50.6)  20 (7.6)  10 (3.8) | <0.001 – 0.96 | 29.2  13.7  48.1  4.4  4.6 | 30.9  17.0  40.4  6.6  5.2 | 0.747 – 0.17 |
| **No. of metastatic sites**  ≤ 2  > 2 | 66 (65.3)  35 (34.7) | 130 (49.4)  133 (50.6) | 0.001 – 0.32 | 49.5  50.5 | 54.0  46.0 | 0.59 – 0.08 |
| **Treatment line of Immunotherapy**  First  Non-First | 47 (46.5)  54 (53.5) | 58 (22.1)  205 (77.9) | <0.001 – 0.53 | 26.0  74.0 | 28.9  71.1 | 0.639 – 0.06 |

**Supplementary Table 5:** IPTW-fitted multivariable analysis for the risk of treatment discontinuation and risk of death including variables with SMD ≥0.1. Patients subsequently entered into PROVIDENCE cohort 2 are included in the control cohort. A centre-specific conditional interpretation by using frailty models was applied to correct all the 95%CIs. HR: hazard ratio; NSCLC: non-small cell lung cancer; ECOG-PS: eastern cooperative oncology group performance status; IPTW: inverse probability of treatment weighing; SMD: standardized mean difference.

|  | **Multivariate Analysis** | |
| --- | --- | --- |
|  | **Risk of Treatment discontinuation** | **Risk of death** |
| **VARIABLE** | **HR (95% CI)** | **HR (95%CI)** |
| **Cohort**  Control  PROVIDENCE cohort 1 | 1  **0.68 (0.47-0.98)** | 1  **0.62 (0.39-0.98)** |
| **ECOG-PS**  0  1  ≥2 | 1  2.03 (1.44-2.86)  4.22 (2.38-7.46) | 1  2.26 (1.43-3.57)  3.80 (1.73-8.33) |
| **Primary Tumour**  NSCLC  Melanoma  Kidney  Urothelial  Others | 1  1.18 (0.82-1.71)  0.82 (0.53-1.24)  0.79 (0.43-1.45)  0.74 (0.33-1.68) | 1  0.88 (0.56-1.37)  0.71 (0.41-1.21)  0.79 (0.39-1.56)  0.76 (0.31-1.87) |
